# Para- and perirenal ultrasonographic fat thickness is associated with 24-hours mean diastolic blood pressure levels in overweight and obese subjects

**DOI:** 10.1186/s12872-015-0101-6

**Published:** 2015-09-30

**Authors:** Giovanni De Pergola, Nicla Campobasso, Adele Nardecchia, Vincenzo Triggiani, Domenico Caccavo, Loreto Gesualdo, Franco Silvestris, Carlo Manno

**Affiliations:** Clinical Nutrition Unit, Medical Oncology, Department of Biomedical Sciences and Human Oncology, Section of Clinical Oncology, University of Bari, School of Medicine, Policlinico, Piazza Giulio Cesare 11, 70124 Bari, Italy; Department of Emergency and Organ Tranplantation (DETO), Section of Nephrology, University of Bari, School of Medicine, Policlinico, Piazza Giulio Cesare, 70124 Bari, Italy; Department of Emergency and Organ Tranplantation (DETO), Section of Endocrinology and Metabolic Diseases, University of Bari, School of Medicine, Policlinico, Piazza Giulio Cesare, 70124 Bari, Italy

**Keywords:** Aldosterone, Ambulatory blood pressure monitoring, Hypertension, Obesity, Para- and perirenal ultrasonographic fat thickness

## Abstract

**Background:**

Renal sinus fat (RSF) has been recognized as a risk factor for arterial hypertension. This study was addressed to examine whether also para- and perirenal fat accumulation is associated to higher 24-h mean systolic (SBP) and/or diastolic blood pressure (DBP) levels in overweight and obese subjects.

**Methods:**

A cohort of 42 overweight and obese patients, 29 women and 13 men, aged 25–55 years, not treated with any kind of drug, was examined. Body mass index (BMI), waist circumference (WC), fasting insulin and glucose serum levels, insulin resistance (assessed by using the homeostasis model assessment [HOMAIR]), and 24-h aldosterone urine levels were measured. Ambulatory blood pressure monitoring (ABPM) was measured with 15 min intervals from 7.0 a.m. to 11.0 a.m. and with 30 min intervals from 23.0 to 7.0 for consecutive 24 h, starting from 8:30 AM. Measurement of para- and perirenal fat thickness was performed by ultrasounds by a duplex Doppler apparatus.

**Results:**

Para- and perirenal ultrasonographic fat thickness (PUFT) was significantly and positively correlated with WC (*p* < 0.01), insulin (*p* < 0.01), HOMAIR (*p* < 0.01), and 24-h mean DBP levels (*p* < 0.05). 24-h mean DBP was also significantly and positively correlated with 24-h aldosterone urine concentrations (*p* < 0.001). A multivariate analysis by multiple linear regression was performed; the final model showed that the association of 24-h mean DBP as dependent variable with PUFT (multiple *R* = 0.34; *p* = 0.026) and daily aldosterone production (multiple *R* = 0.59; *p* = 0.001) was independent of other anthropometric, hormone and metabolic parameters.

**Discussion and Conclusions:**

This study shows a positive independent association between PUFT and mean 24-h diastolic blood pressure levels in overweight and obese subjects, suggesting a possible direct role of PUFT in increasing daily diastolic blood pressure.

## Background

Obesity, and visceral obesity in particular, is associated with higher cardiovascular morbidity and mortality and is recognized as a major public health problem [1, 2]. The classification of obesity using body mass index (BMI) is useful clinically and in epidemiologic studies, but it does not include the biological complexity of fat accumulation. In fact, excess body fat is a heterogeneous condition in which subjects with similar BMI levels may have a different risk of metabolic and cardiovascular diseases [2].

Central fat accumulation provides an explanation for the higher risk of coronary heart disease, that persists after accounting for BMI and common risk factors [3]. Waist circumference is usually considered a clinical mean to quantify the central body fat accumulation, and some clinical guidelines have recommended waist circumference measurement to provide additional information concerning cardiovascular risk [4]. However, waist circumference consists of both subcutaneous adipose tissue and visceral adipose tissue, that is typically ectopic; this factor is important since visceral adipose tissue is related to higher cardiometabolic risk than subcutaneous adipose tissue [5]. Some amount of adipose tissue surrounds several organs and is defined as “ectopic fat”, since this fat is not storaged in classical sites. Interestingly, adipose tissue accumulation in ectopic sites may have systemic and local vascular consequences [6] and clinical consequences.

There are several potential mechanisms that might explain the tendency to deposit adipose tissue in ectopic versus nonectopic depots. One hypothesis suggests that, in states of positive energy balance, excess free fatty acids are initially stored subcutaneously, but once the capacity of subcutaneous adipose tissue is reached, storage shifts to ectopic sites, (viscera, heart, and vasculature) [7]. The failure of subcutaneous adipose tissue to store additional free fatty acids is believed to result from a failure of proliferation and differentiation of adipocytes leading to subcutaneous adipose hypertrophy as opposed to hyperplasia. Ectopic fat depots with predominantly systemic effects include visceral adipose tissue, fatty liver, and intramuscular fat. Ectopic fat depots with potential local effects include pericardial (or the related epicardial or pericoronary fat), myocardial steatosis, and para-, perirenal and renal sinus fat (RSF).

Para- and perirenal fat is that enclosed from the inner side of the abdominal musculature to the surface of the kidney [8]. RSF is a perirenal area bounded from the hilum of the kidney to the edge of the renal parenchyma [9], and is physically separated from the renal parenchyma by a reflection of the external capsule. It constitutes the renal perivascular adipose tissue compartment surrounding the major branches of the renal artery and vein, lymphatic vessels and the major and minor calices of the collecting system and ureters. In animal models, excessive accumulation of fat within the RSF displaces and compresses the low pressure renal lymphatics and veins, as well as the ureters [10]. Compression of these structures increases renal hydrostatic pressure (providing a stimulus to increase renal size) and activates the renin-angiotensin-aldosterone system (RAAS) [10]. Activation of the RAAS promotes hypertension, insulin resistance, atherosclerosis, and other adverse physiological effects related to obesity [10]. Thus, excessive adipose tissue in the RSF could compress low pressure conduits and serve as a stimulus to hypertension, that is associated with cardiovascular events [11]. According to this hypothesis, RSF was found to be associated with hypertension even after adjustment for cardiovascular risk factors [9, 12], including visceral adipose tissue [12]. Interestingly, Chughtai HL et al. showed an independent association between RSF accumulation and the number of medications needed to treat hypertension [9].

As far as we are aware, no study has examined the possibility of a relationship between para- and perirenal fat thickness and daily blood pressure levels in humans and, in particular, in overweight and obese subjects having any pharmacological interference.

We hypothesized that PUFT was associated with higher 24-h mean systolic (SBP) and/or diastolic blood pressure (DBP) in overweight and obese subjects. To address this hypothesis, we measured the association between PUFT and daily blood pressure levels, measured by ambulatory blood pressure measurement (ABPM) in overweight and obese patients without stable hypertension.

The present study was performed in a population of 42 seemingly healthy men and women, either overweight or obese, aged 25–55 years, not using any kind of drug at the moment of enrollment. Anthropometric (BMI, waist circumference), 24-h urine aldosterone levels, fasting glucose and insulin serum levels, and insulin resistance were also measured in the subjects under study.

## Methods

### Subject population

The study subjects were consecutively enrolled at the Outpatient Clinic of Clinical Nutrition, Medical Oncology, Department of Biomedical Sciences and Human Oncology, Section of Clinical Oncology, University of Bari, School of Medicine. They had come to the Outpatient Clinic with the idea of losing weight and receiving advice on diet and lifestyle.

As regards the criteria for inclusion, subjects enrolled had to have a BMI over 25.0, to be older than 18 years and to not be using any kind of drug (including hormone replacement therapy and oral contraception for post-menopausal and pre-menopausal women respectively).

Exclusion criteria were endocrinological diseases, chronic inflammatory diseases, diabetes mellitus, stable hypertension, angina pectoris, stroke, transient ischemic attack, heart infarction and congenital heart disease.

On this basis, respecting the inclusion and exclusion criteria, the study enrolled 42 consecutive patients, represented by 29 women and 13 men, aged 25–55 years.

Informed consent was given verbal by all subjects for the study, and the procedures followed were in accordance with the ethical standards of the responsible committee on human experimentation (institutional and national) and with the Helsinki Declaration of 1975, as revised in 2000. The study was approved by the local ethics committee (Comitato Etico Indipendente Azienda Ospedaliero-Universitaria “Consorziale Policlinico”, Bari), according to a general statement of adherence to standards.

Based on routine blood tests, urinalysis, electrocardiogram, physical examination and medical history, all subjects were judged in good health. None of them had fasting blood glucose levels higher than 126 mg/dl. All subjects had apparent normal function, since all of them showed eGFR > 90 ml/min and none of them showed proteinuria. No participant was on a limited calorie diet nor had been taking part in intense physical activity before enrollment. During the period of testing, all participants agreed to not undertake any sporting activity and to maintain their normal diet. The day prior to measuring, the subjects were requested to abstain from alcohol and caffeine.

#### General data and anthropometric measurements

BMI was calculated as the weight (kg rounded to the nearest kg) divided by the square of height (m rounded to the nearest centimetre). Waist circumference was measured at the anatomic waistline, that should be the narrowest part of the abdomen, which is at the natural indentation between the iliac crest and the 10^th^ rib (minimum waist).

#### Metabolic and hormonal parameters

Blood samples were taken between 8 and 9 am after fasting overnight. Serum insulin concentrations were determined by radioimmunoassay (Behring, Scoppitto, Italy). 24-h urinary collection for measurement of aldosterone levels was collected at home the day before the 24-h blood pressure measurement. 24-h urinary aldosterone concentrations were measured by chemiluminescent immunoassay (LIAISON aldosterone assay, DiaSorin, Saluggia (VC), Italy).

Plasma glucose levels were determined by the glucose-oxidase method (Sclavo, Siena, Italy). Insulin resistance was assessed by using the homeostasis model assessment (HOMAIR) [13].

#### Ambulatory Blood Pressure Measurements (ABPM)

Ambulatory blood pressure was measured with 15 min intervals from 7.0 a.m to 11.0 a.m. and with 30 min intervals from 23.0 to 7.0 for consecutive 24 h, starting from 8:30 AM (Ultralite ABPM Monitor 90217, SpaceLabs Media Inc, Redmond, WA). Heart rate was measured over 24 h by the same instrument.

#### Measurement of renal sinus fat

**Fig. 1:**
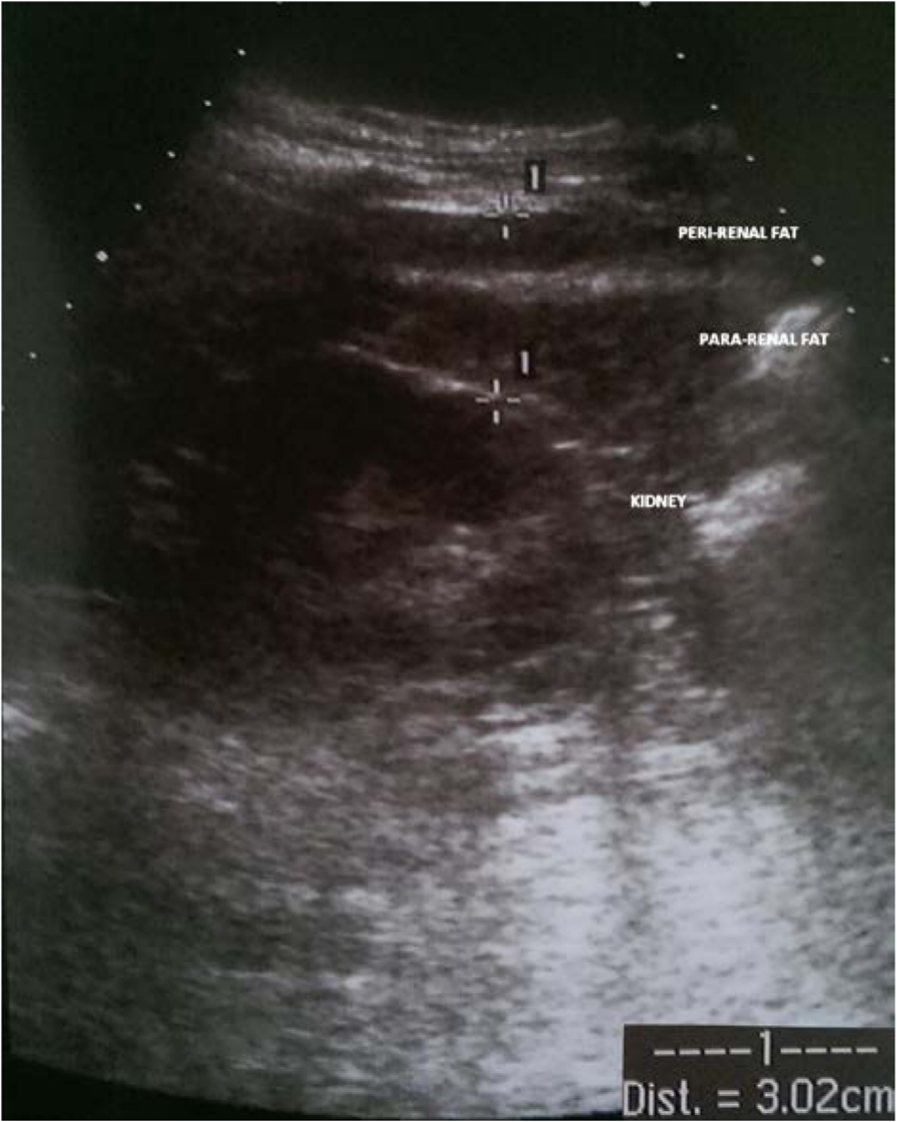
PUFT = para- and perirenal ultrasonographic fat thickness

Measurement of PUFT was performed as previously described by our group [8], and ultrasound examinations were performed by a duplex Doppler apparatus (Acuson Sequoia 512 ultrasound system, Siemens, USA). PUFT was measured with the patient in the supine position. The probe was kept perpendicular to the skin on the lateral aspect of the abdomen. Longitudinal scanning was performed, and the probe was slowly moved laterally until the optimal position was found, at which the surface of the kidney was almost parallel to the skin. The pressure exerted on the probe was as minimal as possible so that the fat layers were not compressed. Then, the ultrasound thickness of para- and perirenal fat was measured from the inner side of the abdominal musculature to the surface of the kidney, and the average of the ultrasound measurement of the maximal thickness values on both sides was defined as the PUFT. The correlation between PUFT values measured on both sides was 0.749 (*P* < 0.0001). PUFT was measured three times. The intraoperator coefficient of variation was 4.5 %. Sonographer (N.C.) was blinded to any other aspect of the study. An ultrasound image of PUFT is shown in Fig. 1.

#### Statistical analysis

Results are expressed as mean ± standard deviation. To test the significant independent relationship between PUFT or 24-h mean diastolic blood pressure and anthropometric, hormone and metabolic parameters we determined Pearson correlation coefficient, since all the data were normally distributed. Furthermore, to test the independent relationship between 24-h mean DBP and the other examined parameters we constructed multivariate models by multiple linear regression analysis based on 24-h mean diastolic blood pressure (dependent variable) and significant variables by univariate analysis; clinically significant variables were forced in the models if *P* ≤ 0.20. Significant independent variables were identified by a stepwise approach. By this approach, we constructed a final model of adequate statistical power (at least 15 subjects for each variable). Data are expressed as unstandardized (B) and standardized (β) regression coefficient. All analyses were performed using SPSS for Windows, release 17.0 (SPSS, Inc., North Sydney, Australia); *P <* 0.05 was considered statistically significant.

## Results

The general, anthropometric, metabolic and hormone parameters from the study population are shown in Table 1. The mean value of PUFT in 42 overweight or obese patients (BMI 33.3 ± 4.3 Kg/m^−2^) was 25.0 ± 8.1 cm. The 24-h mean DBP was 82.4 ± 8.5 mmHg.

**Table 1:** General, anthropometric, metabolic and hormone parameters in subjects studied (*n* = 42)

Age (years)	44.8 ± 9.3
Body mass index (kg/ m^−2^)	33.3 ± 4.3
Waist circumference (cm)	108.5 ± 11.2
Fasting blood glucose (mg/dl)	87.3 ± 8.3
Fasting insulin (μUI/ml)	16.0 ± 8.1
HOMAIR	3.48 ± 1.91
24-h urine aldosterone (μg/24 h)	16.1 ± 6.64
Office systolic BP (mmHg)	137.0 ± 11.3
Office diastolic BP (mmHg)	88.3 ± 8.9
24-h mean systolic BP (mmHg)	131.8 ± 10.5
24-h mean diastolic BP (mmHg)	82.4 ± 8.5
Diurnal systolic BP (mmHg)	135.8 ± 10.5
Diurnal diastolic BP (mmHg)	85.8 ± 8.5
Nocturnal systolic BP (mmHg)	120.7 ± 13.5
Nocturnal diastolic BP (mmHg)	73.3 ± 9.9
PUFT (mm)	25.0 ± 8.1

Data are expressed as mean ± standard deviation

*PUFT* para- and perirenal ultrasonographic fat thickness

**Fig. 2:**
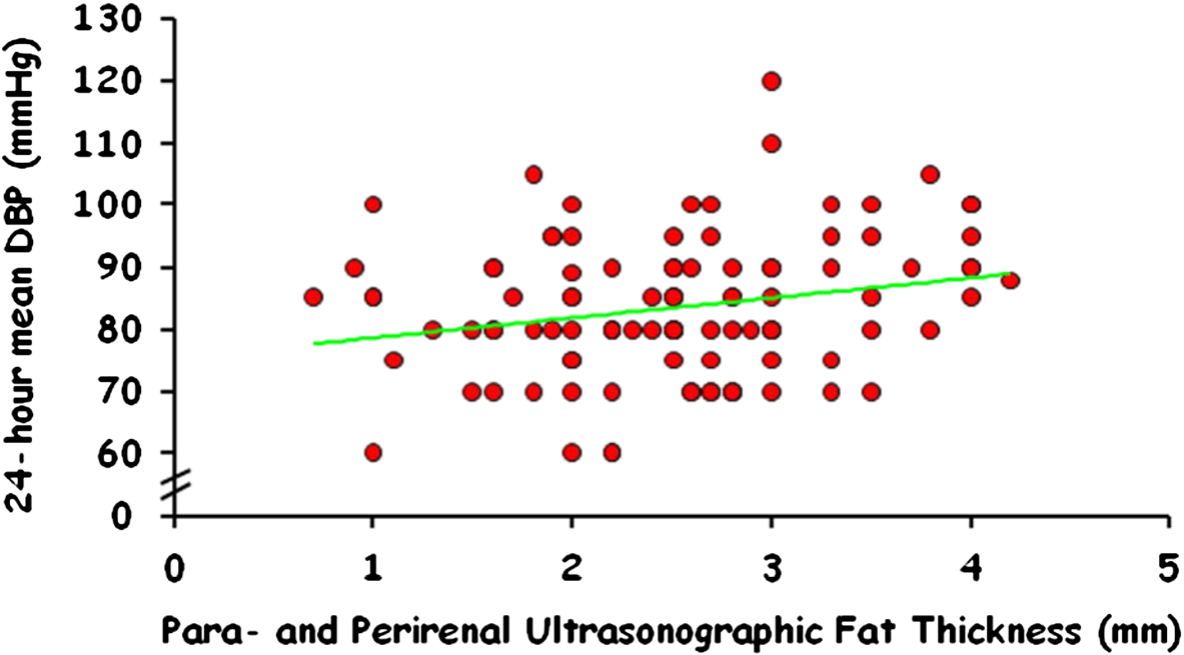
Correlation between PUFT and 24-h mean diastolic blood pressure

Table 2 show the correlations between RSF and all other parameters in 42 subjects under study. The RSF was significantly and positively correlated with waist circumference (*r* = 0.39; *p* = 0,01), insulin (*r* = 0.45; *p* < 0.003), HOMAIR (*r* = 0.45; *p* < 0.003), and 24-h mean DBP levels (*r* = 0.34; *p* = 0.026) (Fig. 2).

**Table 2:** Correlations between PUFT (mm) and anthropometric, hormone and metabolic parameters in subjects under study (*n* = 42)

Parameter	*r*	*p* value
Age (years)	0.10	0.545
Body mass index (kg/ m^−2^)	0.11	0.498
Waist circumference (cm)	0.39	0.010
Fasting blood glucose (mg/dl)	0.10	0.514
Fasting insulin (μUI/ml)	0.45	0.003
HOMAIR	0.45	0.003
Office systolic BP (mmHg)	0.03	0.874
Office diastolic BP (mmHg)	0.20	0.199
24-h urine aldosterone (μg/24 h)	0.32	0.040
24-h systolic BP (mmHg)	0.01	0.976
24-h diastolic BP (mmHg)	0.34	0.026
Diurnal systolic BP (mmHg)	0.01	0.932
Diurnal diastolic BP (mmHg)	0.29	0.059
Nocturnal systolic BP (mmHg)	0.11	0.504
Nocturnal diastolic BP (mmHg)	0.20	0.205

*r* represents the Pearson correlation coefficient

*PUFT* para- and perirenal ultrasonographic fat thickness

No correlation was found between 24-h mean SBP and anthropometric, hormone and metabolic parameters in subjects under study, except for 24-h mean DBP (*r* = 0.80; *p* = 0.001), diurnal SBP (*r* = 0.96; *p* = 0.001), diurnal DBP (*r* = 0.76; *p* = 0.001), nocturnal SBP (*r* = 0.81; *p* = 0.001) and nocturnal DBP (*r* = 0.70; *p* = 0.001). Furthermore, 24-h mean SBP directly correlated with 24-h urine aldosterone levels (*r* = 0.44; *p* = 0.004), but not with RSF (*r* = 0.01; *p* = 0,976).

**Fig. 3:**
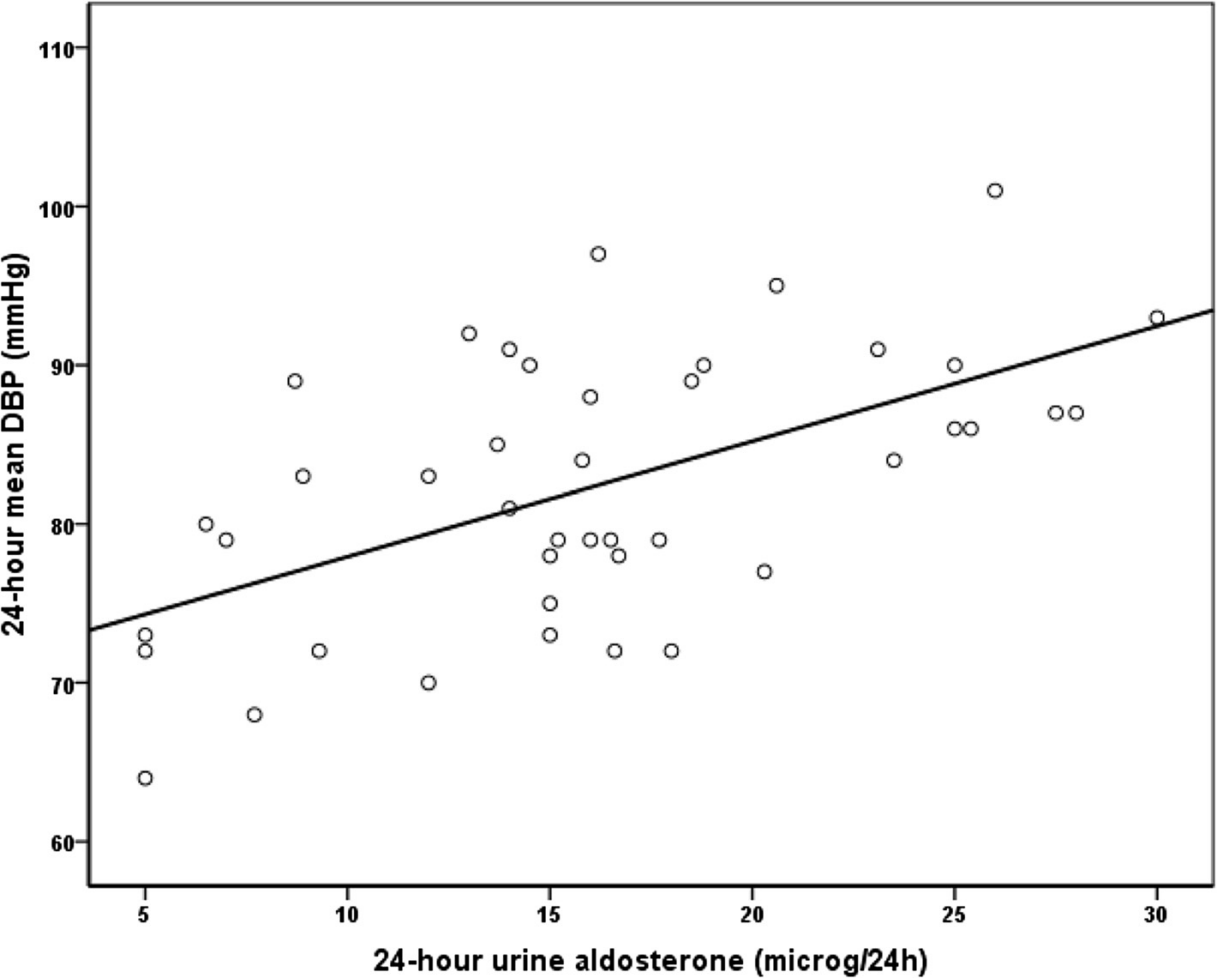
Correlation between 24-h mean diastolic blood pressure and 24-h urine aldosterone

Table 3 shows the correlations between 24-h mean DBP levels and all other parameters in 42 subjects. The 24-h mean DBP was significantly and positively correlated with 24-h urine aldosterone levels (*r* = 0.57; *p* <0.001) (Fig. 3), 24-h mean SBP (*r* = 0.80; *p* <0.001), diurnal mean SBP (*r* = 0.76; *p* <0.001) and DBP (*r* = 0.96; *p* <0.001), and nocturnal mean SBP (*r* = 0.69; *p* < 0.001) and DBP (*r* = 0.81; *p* < 0.001). No correlation was found between mean DBP and office SBP or DBP.

**Table 3:** Correlations between 24-h diastolic blood pressure (mmHg) and anthropometric, hormone and metabolic parameters in subjects under study (*n* = 42)

Parameter	*r*	*p* value
Age (years)	0.09	0.588
Body mass index (kg/m^−2^)	0.12	0.458
Waist circumference (cm)	0.23	0.138
Fasting blood glucose (mg/dl)	0.09	0.548
Fasting insulin (μUI/ml)	0.22	0.172
HOMAIR	0.21	0.183
24-h urine aldosterone (μg/24 h)	0.57	< 0.001
Office systolic BP (mmHg)	0.16	0.311
Office diastolic BP (mmHg)	0.23	0.149
24-h mean systolic BP (mmHg)	0.80	< 0.001
Diurnal systolic BP (mmHg)	0.76	< 0.001
Diurnal diastolic BP (mmHg)	0.96	< 0.001
Nocturnal systolic BP (mmHg)	0.69	< 0.001
Nocturnal diastolic BP (mmHg)	0.81	< 0.001
PUFT (mm)	0.34	0.026

*r* represents the Pearson correlation coefficient

*PUFT* para- and perirenal ultrasonographic fat thickness

As shown in Table 4, multivariate analysis by multiple linear regression confirmed that the association of 24-h mean diastolic blood pressure (dependent variable) and PUFT (multiple *R* = 0.34; *p* = 0.026) and 24-h urine aldosterone levels (multiple *R* = 0.59; *p* = 0.001) was independent of other variables added to the models (waist circumference, body mass index); obviously, we did not add any other measure of blood pressure. Indeed, in the final model, PUFT ranked as the second correlate 1 (β = 0.17) of 24-h mean DBP, while the strong independent correlate was daily aldosterone production (β = 0.51).

**Table 4:** Multiple linear regression between 24-h mean diastolic blood pressure (dependent variable) and potential predictors parameters in 42 subjects under study

Parameter	Unstandardized	Standardized	Multiple R	Multiple R2	*p* value
	Coefficient (B)	Coefficient β			
PUFT	1.73	0.17	0.34	0.12	0.026
24 hour urine aldosterone levels	0.65	0.51	0.59	0.35	< 0.001

*PUFT* para- and perirenal ultrasonographic fat thickness

## Discussion

This study, performed in overweight and obese otherwise healthy subjects, shows a direct independent relationship between 24-h diastolic blood pressure (as measured by ambulatory blood pressure measurement) and both para- and perirenal fat thickness (measured by ultrasounds) and daily aldosterone production.

To the best of our knowledge, this is the first evidence demonstrating that PUFT is a powerful independent predictor of higher 24-h mean DBP levels.

It has been shown that compression of renal artery and vein, and lymphatic vessels, increases renal hydrostatic pressure (providing a stimulus to increase renal size) and activates RAAS, with higher 24-h urine aldosterone levels [10]. Even though daily urine aldosterone was a strong predictor of 24-h mean DBP levels in this study, the correlation between PUFT and 24-h mean DBP levels was maintained after adjusting for daily urine aldosterone levels, suggesting that PUFT may independently influence 24-h mean DBP. It may well be that PUFT contributes to increase renal hydrostatic pressure and/or the production of adipokines with vasoconstrictor effects, thus increasing diastolic more than systolic blood pressure. The interest of these results is emphasized by the fact that only subjects who were not taking any kind of drug were enrolled into this study.

The direct independent relationship between 24-h mean DBP levels and daily aldosterone urine concentrations is in line with previous studies showing that BMI predicts aldosterone concentrations in overweight-obese primary hypertensive patients [14].

Fasting insulin and glucose levels and insulin resistance (measured by HOMAIR) did not show a significant relationship 24-h mean SBP or DBP levels. These results are in line with our previous study performed in overweight and obese patients, normotensive or with recently developed hypertension, never treated with antihypertensive drugs, showing that insulin and HOMAIR were not significantly different between normotensive and hypertensive subjects and were not associated to hypertension or to 24-h mean SBP or DBP levels [15]. Actually, there is still debate on whether insulin resistance is a cause or a consequence of hypertension or whether both conditions arise from a common substrate [15].

Central fat accumulation (as measured by waist circumference), but not BMI, showed a significant correlation with PUFT, strongly suggesting that PUFT progressively increases with abdominal fat accumulation.

On the other hand, both BMI and waist circumference did not show a significant association with 24-h mean SBP or DBP levels in this study. These findings suggest that PUFT *per se* is more important that BMI or central fat accumulation for diastolic blood pressure. Moreover, these results are again in line with our previous study, showing that BMI and waist circumference were not significantly different between normotensive and hypertensive subjects [15].

Office systolic and diastolic blood pressure did not show a significant association with PUFT and 24-h mean SBP or DBP levels, suggesting that office blood pressure measurements are a weak way to evaluate risk factors for hypertension or to perform the diagnosis of hypertension.

PUFT was significantly and positively correlated with insulin and HOMAIR, suggesting that para- and perirenal fat accumulation is paralleled by a progressive increase in insulin resistance and insulin levels.

Concerning the possible interest of the present study, the relationship between RSF and blood pressure was already known [9], but the novelty introduced by this study was in both the evaluation of 24-h blood pressure and the exclusion of subjects having any pharmacological interference. As far as the limitations of the present study is concerned, ultrasounds, and not computed tomography (CT), were used. However, we are going to perform a population study, and it is not easy to use CT scan in population studies. Second, this is a pilot study performed on a relative small number of overweight and obese outpatient subjects and, therefore, it was not possible to plan a sample size for a descriptive study. However, we did not find any previous study showing an independent relationship between PUFT and 24-h blood pressure in the literature, and we believe that the information deriving from the present study may be useful to start a descriptive study with a higher number of patients.

## Conclusions

In conclusion, this study, performed in overweight or obese otherwise healthy subjects, shows a positive association between PUFT and mean daily diastolic blood pressure, independently of anthropometric, hormone and metabolic parameters. Our findings suggest that measurement of PUFT may represent a helpful parameter to evaluate the risk of developing hypertension in overweight and obese subjects.

## Abbreviations

RSF: Renal sinus fat
ABPM: Ambulatory blood pressure monitoring
SBP: Systolic blood pressure
DBP: Diastolic blood pressure
BMI: Body mass index
WC: Waist circumference
HOMAIR: Homeostasis model assessment
RAAS: Renin-angiotensin-aldosterone system
PUFT: Para- and perirenal ultrasonographic fat thickness
